# Supplementary material for: Structural basis for dolichylphosphate mannose biosynthesis
Source: Nat Commun. 2017 Jul 25;8:120. doi: 10.1038/s41467-017-00187-2 (PMC5526996; doi:10.1038/s41467-017-00187-2)
Supplement: Supplementary file 1 — Supplementary information [file 41467_2017_187_MOESM1_ESM.pdf]

File Name: Supplementary Information
Description: Supplementary Figures, Supplementary Tables and Supplementary Referemces

File Name: Peer Review File
Description:

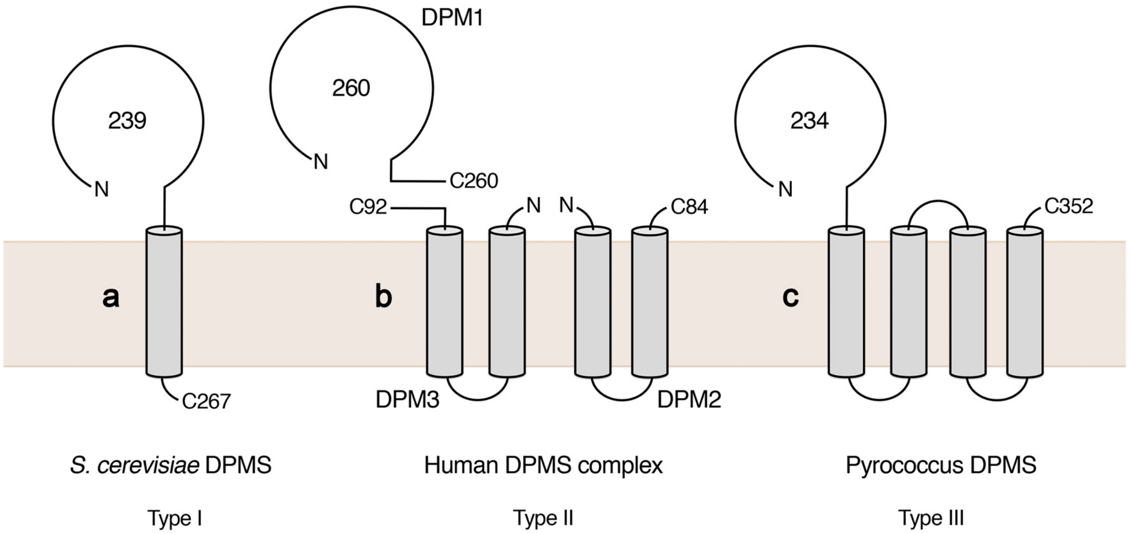

Supplementary Figure 1 | Comparison of DPMS topology. *S. cerevisiae* type-I DPMS, human DPMS type-II complex with three separately encoded subunits DPM1, DPM2 and DPM3[1], and the type-III Pyrococcus enzyme encoded as a single polypeptide chain.

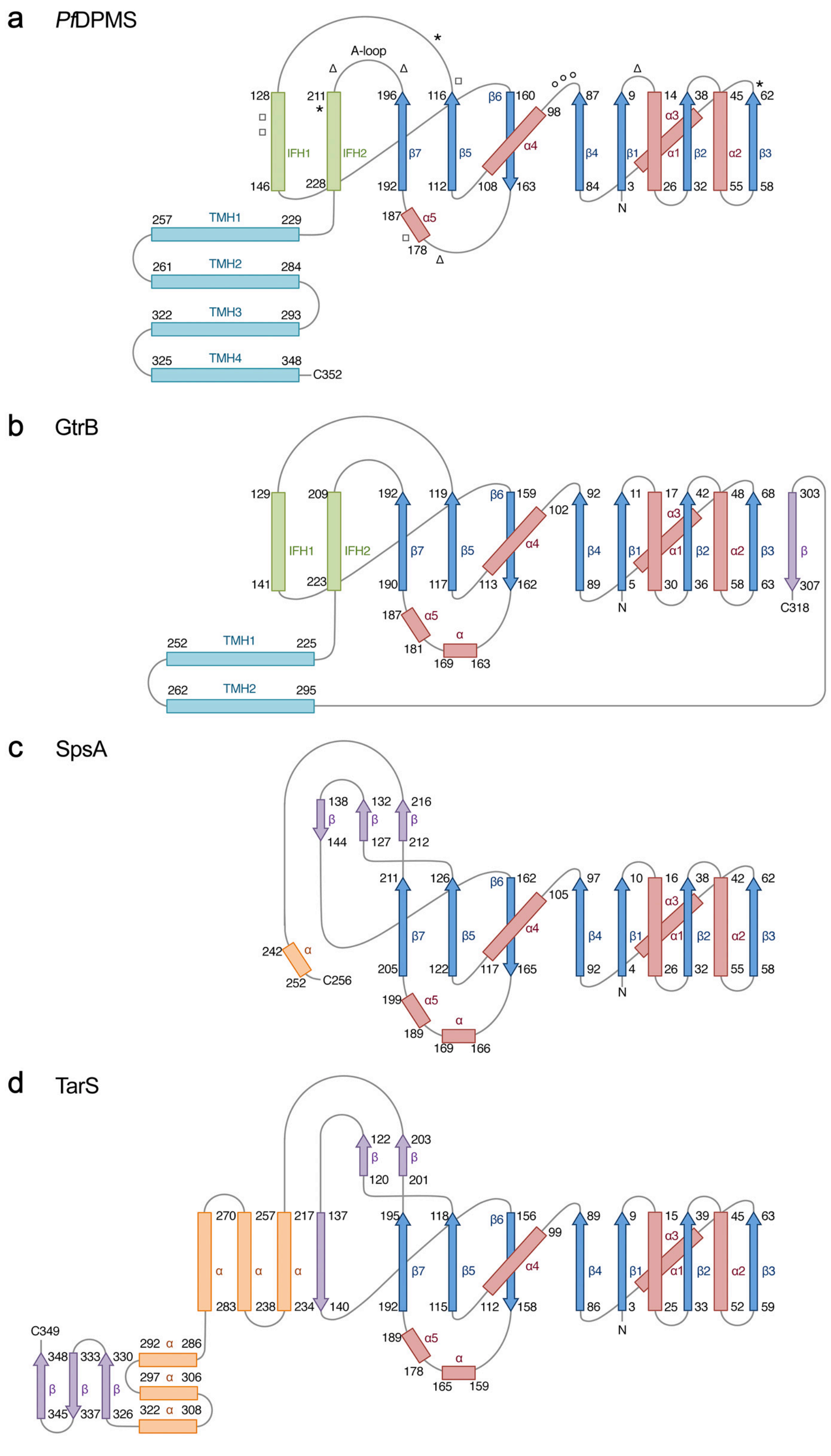

Supplementary Figure 2 | Comparison of the GT-A fold in related GT2 enzymes. Topology diagrams for the membrane GT2 enzymes (**a**) *Pf*DPMS and (**b**) *Synechocystis* GtrB, and for two soluble GT2 members (**c**) *B. subtilis* spore coat protein SpsA, and (**d**) *S. aureus* TarS. For the

canonical GT-A fold, α-helices are shown as red rectangles and β-strands as dark blue arrows. Additional protein-unique secondary-structure elements are colored as follows: α-helix, orange; β-strand, purple; IF helix, green; and TM helix, cyan. For clarity, β-strands comprising fewer than three amino acids, helical turns, and the numbering for α3 have been omitted. Only elements that are part of the GT-A fold are numbered. Relevant amino acids and structural features in *Pf*DPMS discussed in the text are highlighted in the diagrams: open circles, donor and metal-binding residues in the extended DXD motif (Asp89, Asp91 and Gln93); open squares, side chains that participate in recognition of the Dol-*P*-Man phosphate group (Arg117, Arg131, Ser135 and Lys178); open triangles, side chains that secure the acceptor loop in the closed conformation (Glu12, Arg202, Lys208) and Phe177 that gates the "back door" of the donor-binding pocket; asterisks, side chains that are associated with CDG-Ie mutations in human DPM1.

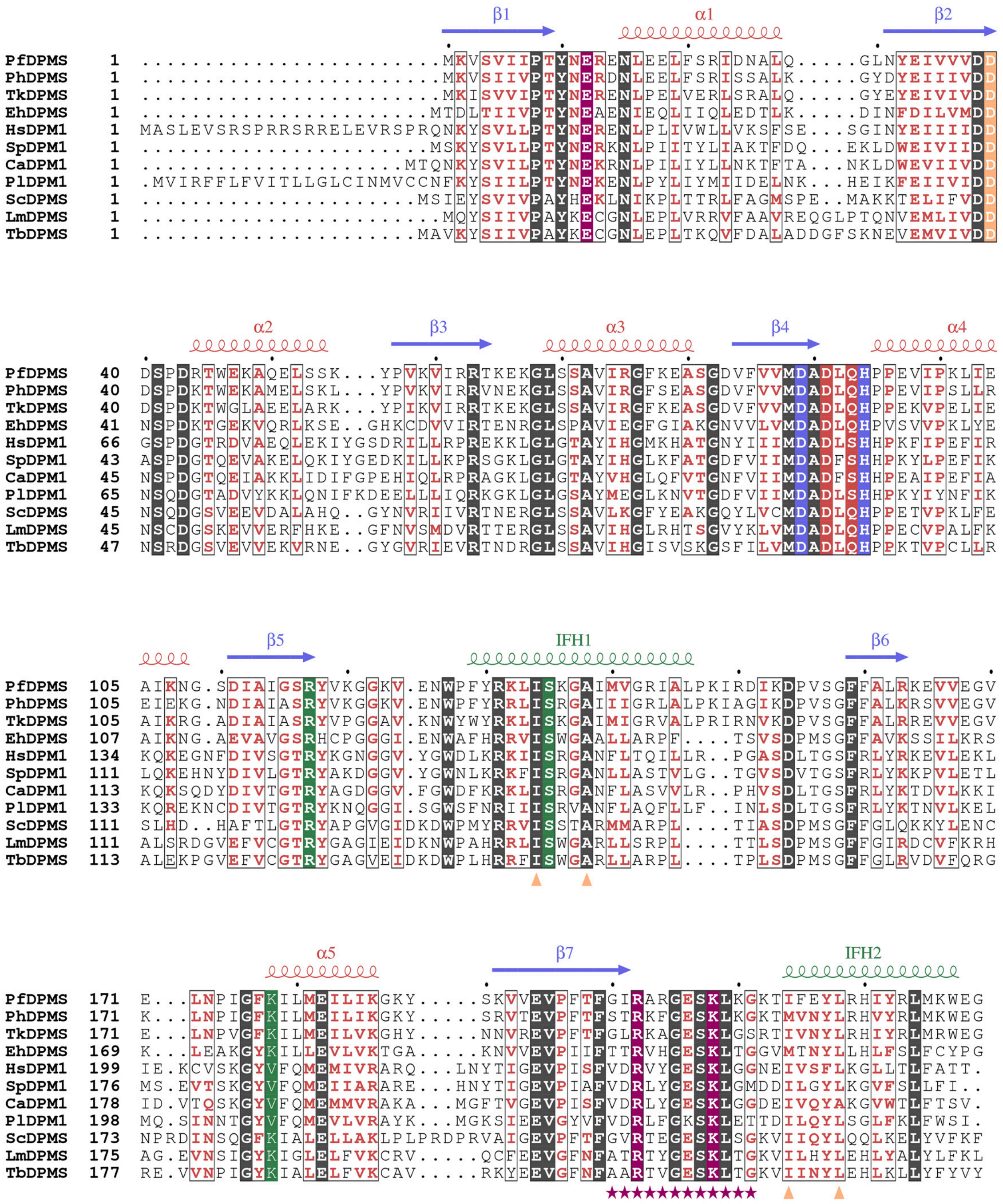

Supplementary Figure 3 | Sequence alignment of type-I, -II and -III catalytic DPMS domains. Alignment of the catalytic domains (DPM1) for selected representatives from the different types of DPMSs: type-III, *Pf*DPMS, *Ph*DPMS, *Tk*DPMS; type-II, *Sp*DPM1, *Ca*DPM1, *Pl*DPM1; type-I, *Sc*DPMS, *Lm*DPMS, *Tb*DPMS; and unclassified, *Eh*DPMS. UniProt accession numbers: *Pf*DPMS, *Pyrococcus furiosus* (Q8U4M3); *Ph*DPMS, *Pyrococcus horikoshii* (O57812); *Tk*DPMS, *Thermococcus kodakarensis* (Q5JES4); *Hs*DPM1, *Homo sapiens* (O60762); *Sp*DPM1, *Schizosaccharomyces pombe* (O14466); *Ca*DPM1, *Candida albicans* (Q5A789); *Pl*DPM1, *Plasmodium falciparum* (Q8IHU9); *Sc*DPMS, *Saccharomyces cerevisiae* (P14020); *Lm*DPMS, *Leishmania mexicana* (O96795); *Tb*DPMS, *Trypanosoma brucei* (Q5QQ41); *Eh*DPMS,

Entamoeba histolytica HM-1:IMSS-A (N9V739). Dark gray boxes with bold white text = strict identity; box frames in dark gray = global similarity; red bold chars = group similarity; bold characters = global similarity; regular characters = low global similarity; purple-shaded boxes = residues that secure the closed acceptor loop (E12, R202, K208); green-shaded boxes = residues that coordinate diphosphate in Dol-*P* and Dol-*P*-Man; blue-shaded boxes = mannosyl-binding binding residues (D89, H94); red-shaded boxes = metal-binding residues (D91, D93); orange-shaded boxes = guanosine-binding residue (D39); blue arrows = β strands; red spirals = α helices; purple stars = acceptor loop; orange triangles = side chains that interact with the first two isoprene units of Dol-*P*-Man (IFH1, I134 and A138; and IFH2, I214 and L218). *Eh*DPMS has a four-transmembrane domain and is the eukaryotic member that is most similar to *Pf*DPMS with respect to membrane topography. To account for the function of the DXD motif in DPMSs, the motif can be expanded to $DADX_1X_2H$ (^{89}DADLQH^{94} in *Pf*DPMS); where the alanine is invariant, X_1 is hydrophobic (Leu or Phe), X_2 is Gln or Ser, and the His is invariant. The two interface helices IFH1 and IFH2 in *Pf*DPMS comprise residues 129-146 and 213-227, respectively.

The enzyme AglD, proposed to catalyze synthesis of the phosphodolichol-linked mannose that serves as mannose pool for the final step in *N*-glycosylation of the S-layer glycoprotein in *Haloferax volcanii*[2,3] has not been included in the sequence alignment. While the activity of AglD indicates a functional kinship to *bona fide* DPMSs, differences at the sequence level precludes assignment of *Pf*DPMS as a member of the AglD subfamily of GT2 enzymes: the sequence identity between *Pf*DPMS and AglD is only 24%, and AglD contains eight predicted transmembrane helices compared with four in *Pf*DPMS. Furthermore, several of the amino acids that are important for Dol-*P*-Man synthesis in true DPMSs appear to be absent in AglD. Based on sequence similarity, the *Hfx. volcanii* gene *DPM1L_HALVD* appears more closely related to *Pf*DPMS (31% sequence identity to the catalytic domains) than AglD, but this gene product has so far not been characterized.

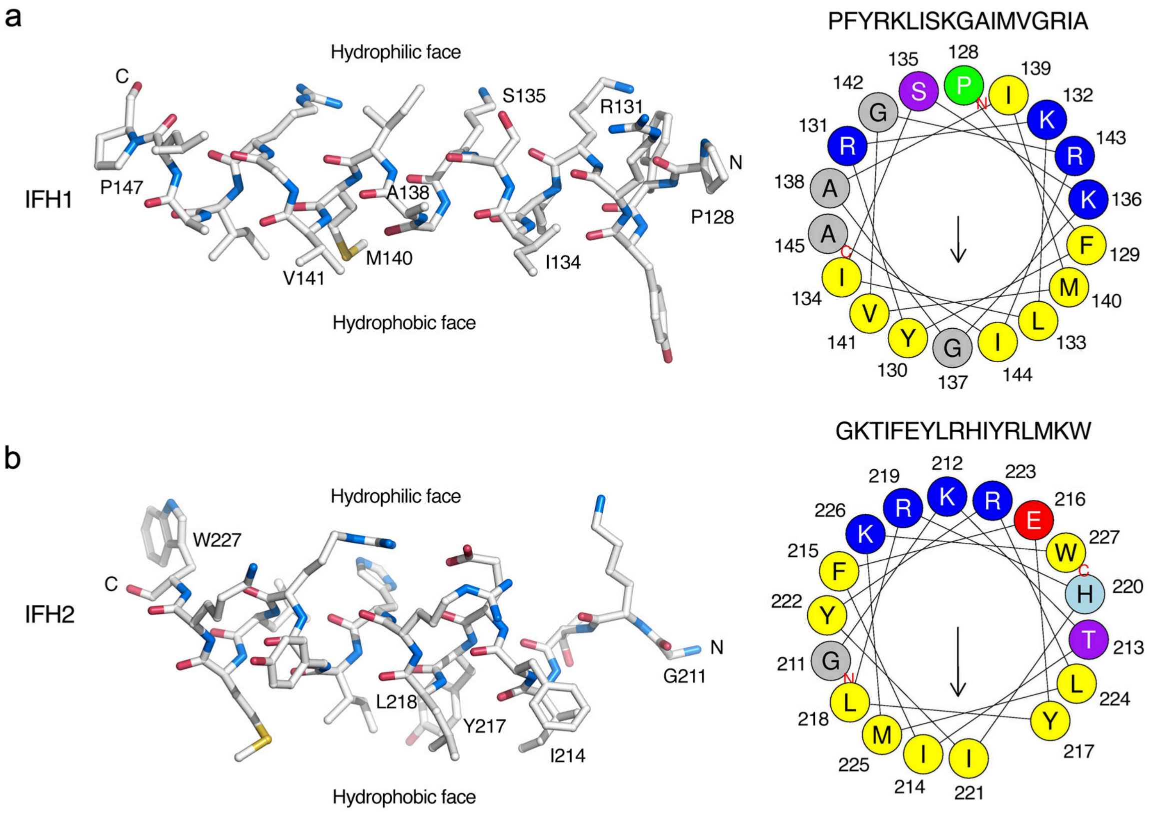

Supplementary Figure 4 | Amphipathicity of the interface helices. Distribution of amino acids in the interface helices (**a**) IFH1 and (**b**) IFH2. The hydrophobic sides are facing the TM domain and the hydrophilic faces pack against the GT-A domain. Side chains that coordinate the phosphate group in Dol-*P*-Man are marked (Ser135 and Arg131), as well as the side chains in IFH1 (Ile134, Ala138, Met140, Val141) and IFH2 (Ile214, Tyr217, Ile218) that interact with the first two isoprene units of the dolichyl chain. The helical wheel diagrams were calculated using heliQuest (http://heliquest.ipmc.cnrs.fr[4]). Color scheme for side chains plotted in the helical wheels: positively charged, dark blue; negatively charged, red; hydrophobic, yellow; polar, green, purple and cyan; small, gray.

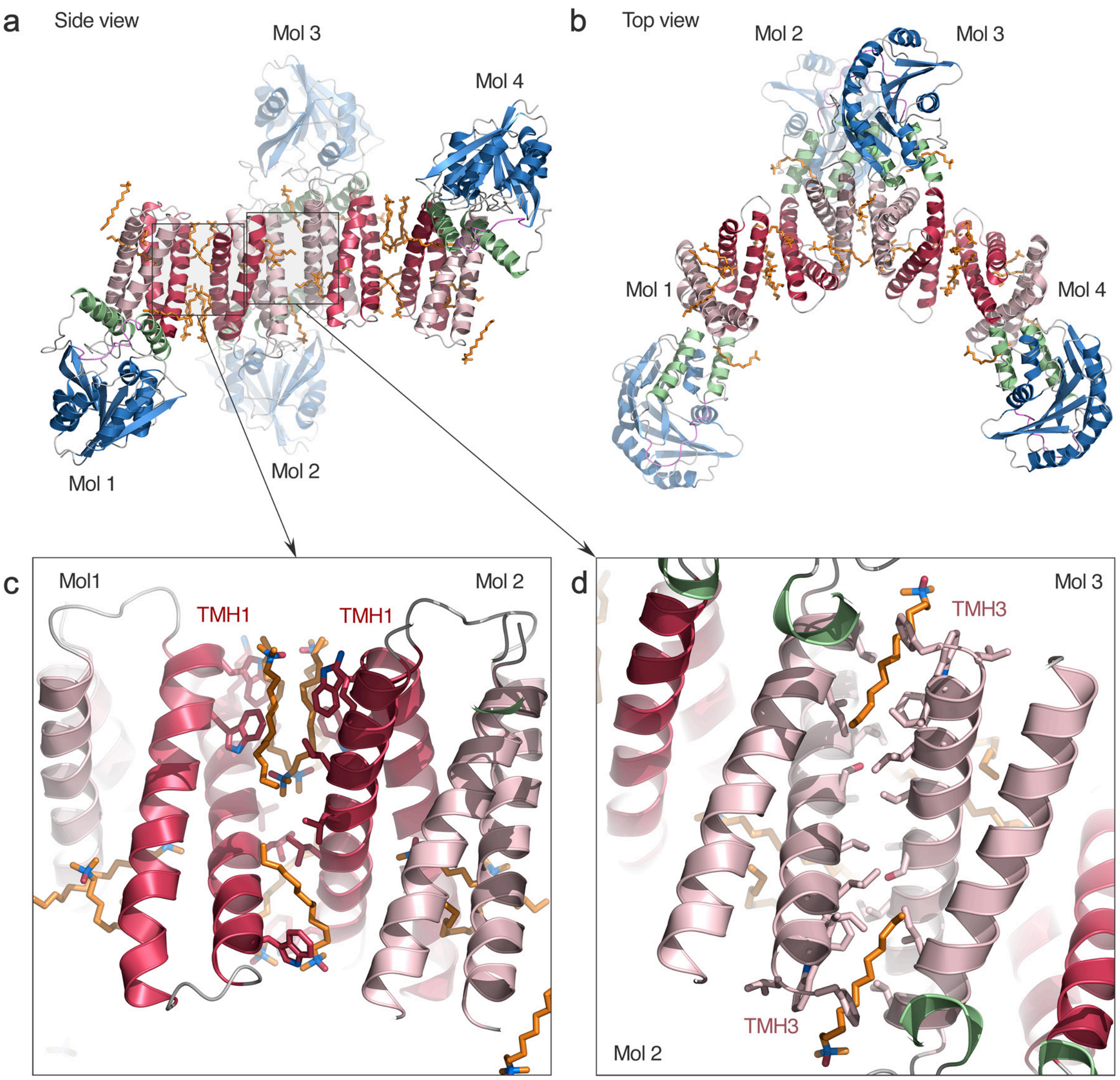

Supplementary Figure 5 | Crystal packing of *Pf*DPMS molecules in the *C*222$_1$ crystal. Side view (**a**) and top view (**b**) of the TM domains in neighboring, crystallographically related *Pf*DPMS molecules. The catalytic domain is colored blue, IF helices green, TMD1 red and TMD2 pink. Two different TMD interfaces are formed between molecules in the crystal lattice, (**c**) TMH1-TMH1 and (**d**) TMH3-TMH3. LDAO molecules interacting with the TM domains are shown in orange.

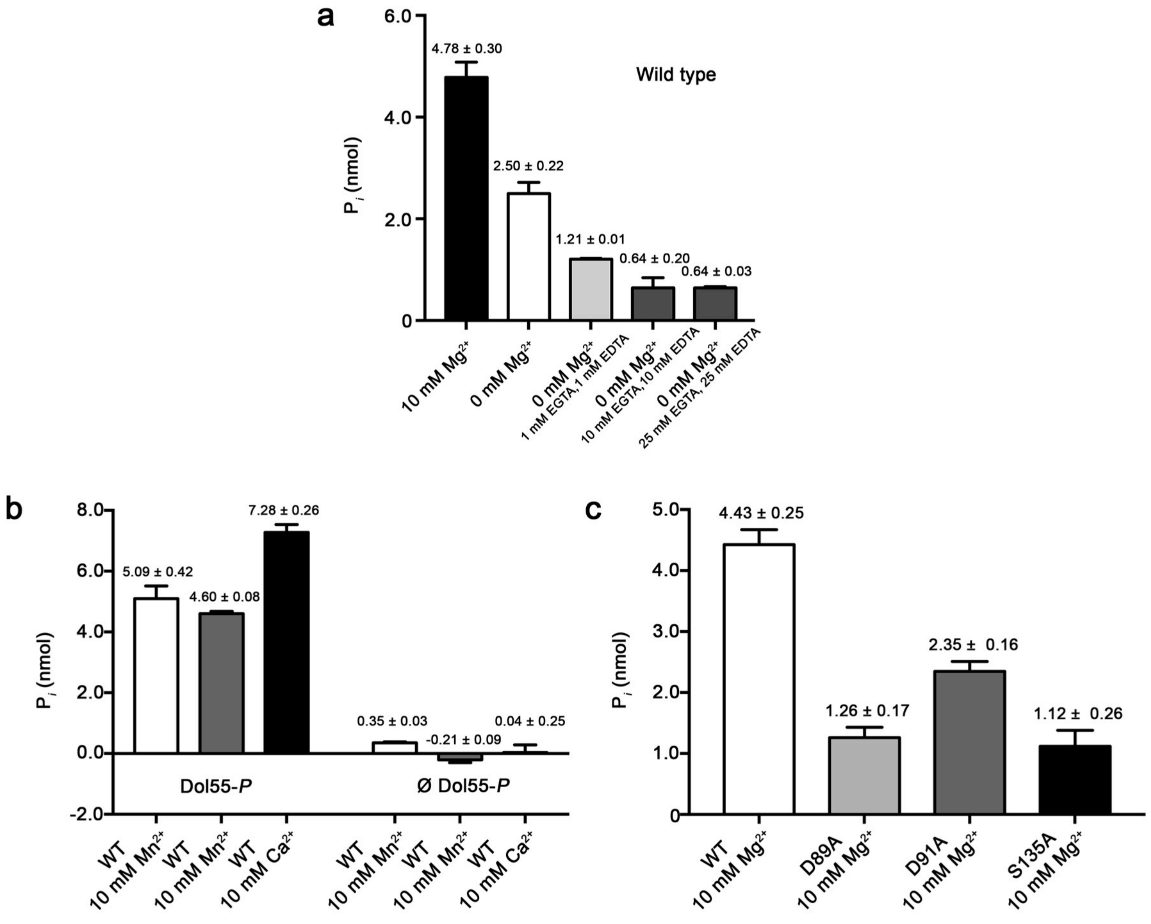

Supplementary Figure 6 | Dependency on metal ion. (**a**) Control experiment showing the level of catalytic activity for wild-type *Pf*DPMS in the presence and absence of metal ion, and in the absence of metal ion with increasing concentration of EGTA and EDTA. For all samples, *Pf*DPMS had also been treated with EDTA and EGTA prior to the experiment as described in the Methods section. Errors are given as mean values ± SEM (*N*=9 for WT and WT without metal; for the rest, *N*=4). (**b**) Activity of wild-type *Pf*DPMS in the presence of different divalent cations. Errors are given as mean values ± SEM (*N*=3). (**c**) Catalytic activity of wild-type *Pf*DPMS, the alanine replacements of Asp89 and Asp91 in the DXD motif, and of Ser135 (responsible for coordinating the phosphate group in the acceptor lipid). The activity is measured as nanomoles of released free phosphate. Errors are given as mean values ± SEM (*N*=7 for WT and *N*=3 for the rest).

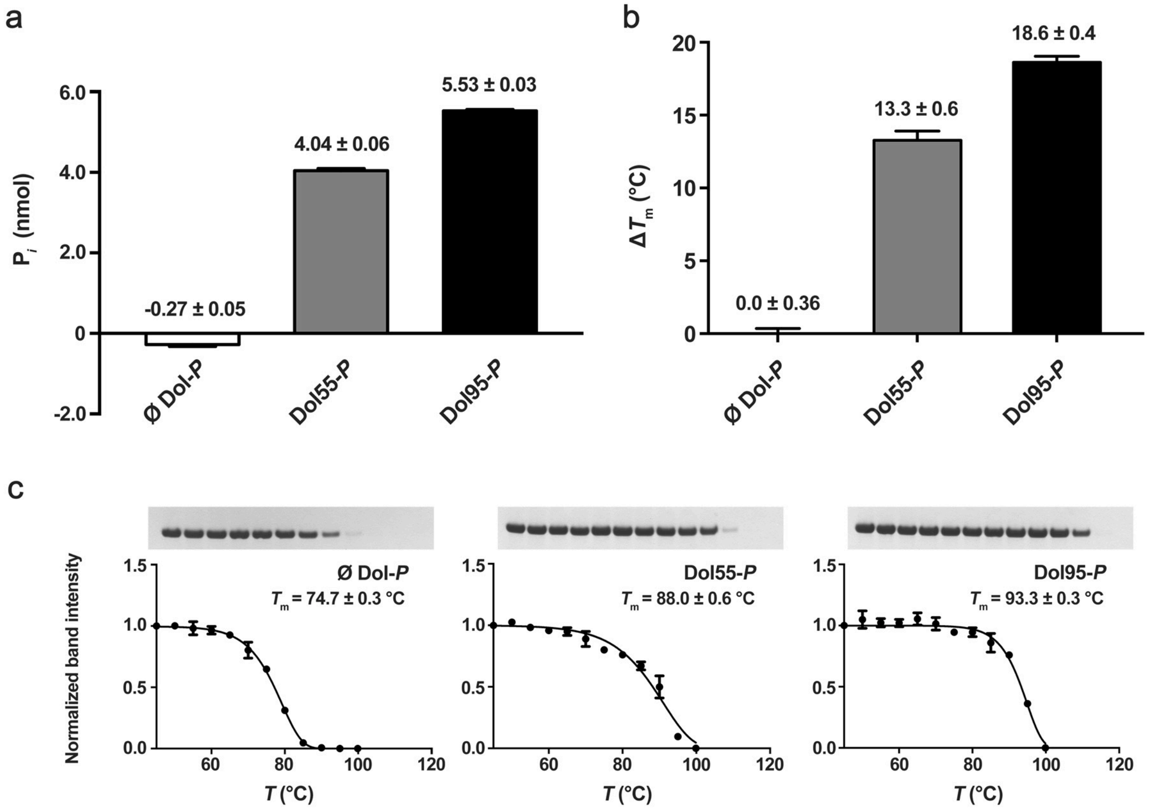

Supplementary Figure 7 | Influence of dolichyl chain length on activity and stability. Assessment of the effect of Dol-*P* acceptor chain length, 55 or 95 carbon atoms, on *Pf*DPMS activity and thermal stability to unfolding. (**a**) Activity measured as nanomoles of released free phosphate as a function of acceptor isoprenoid chain length. The assay included 0.625 nmol enzyme, 10 nmol GDP-Man and 11 nmol Dol55-*P* or 7 nmol Dol95-*P*. Errors are given as mean values ± SEM (*N*=3). (**b**) Increase in melting temperature (ΔT_m) based on the TSA denaturation curves in (**c**). The assay included 6.5 nmol enzyme and ~100 nmol Dol55-*P* or Dol95-*P*. For (b), errors are given as mean values ± SEM (*N*=2), and for (c), errors are given as mean values ± STDEV (*N*=2).

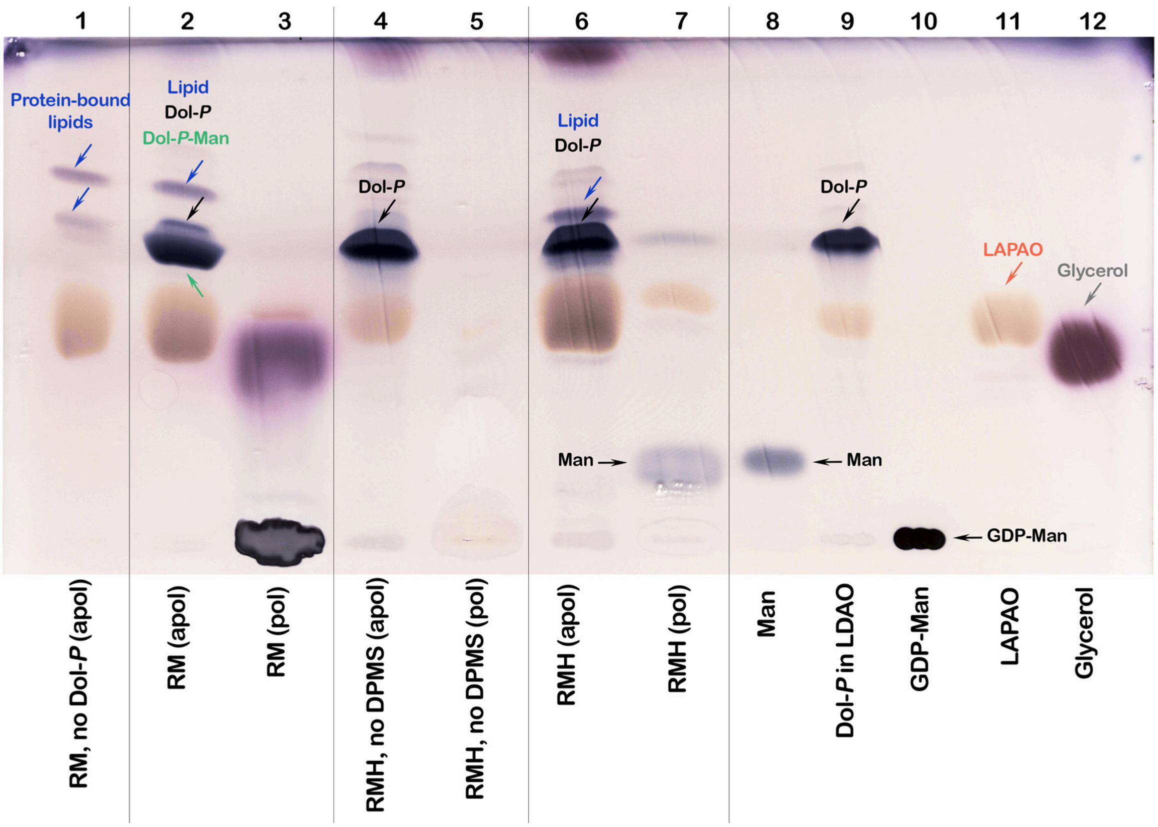

Supplementary Figure 8 | TLC results of Dol95-*P*-Man synthesis. See text for details regarding the TLC running conditions. Samples 1, 4-5 and 8-12 are controls. Loaded samples: (1) a reaction mixture (RM) without added Dol95-*P* acceptor (blue arrows show lipids retained by the protein); (2) apolar and (3) polar extraction phases of RM (half of the total reaction volume of 40 µL was loaded for each sample). Dol95-*P* and Dol95-*P*-Man co-migrate with the Dol95-*P*-Man spot positioned slightly below the Dol95-*P* spot. To verify Dol95-*P*-Man, acid hydrolysis was performed on a control sample, identical to sample 2 but without DPMS, and on the RM sample 2. The hydrolyzed control and RM samples where again extracted into apolar and polar phases (4 and 5 for the control, and 6 and 7 for RM sample 2). The presence of Dol95-*P*-Man in sample 2 is evidenced by release of mannose, whereas no free mannose is produced for the control; (8) 3.5 µL 5 mM D-mannose; (9) 3 µL Dol95-*P* (in 50 mM HEPES pH 7.5, 150 mM NaCl, 0.07% LAPAO); (10) 3 µL 100 mM GDP-Man; (11) 9 µL 1% LAPAO; (12) 6 µL 5% glycerol.

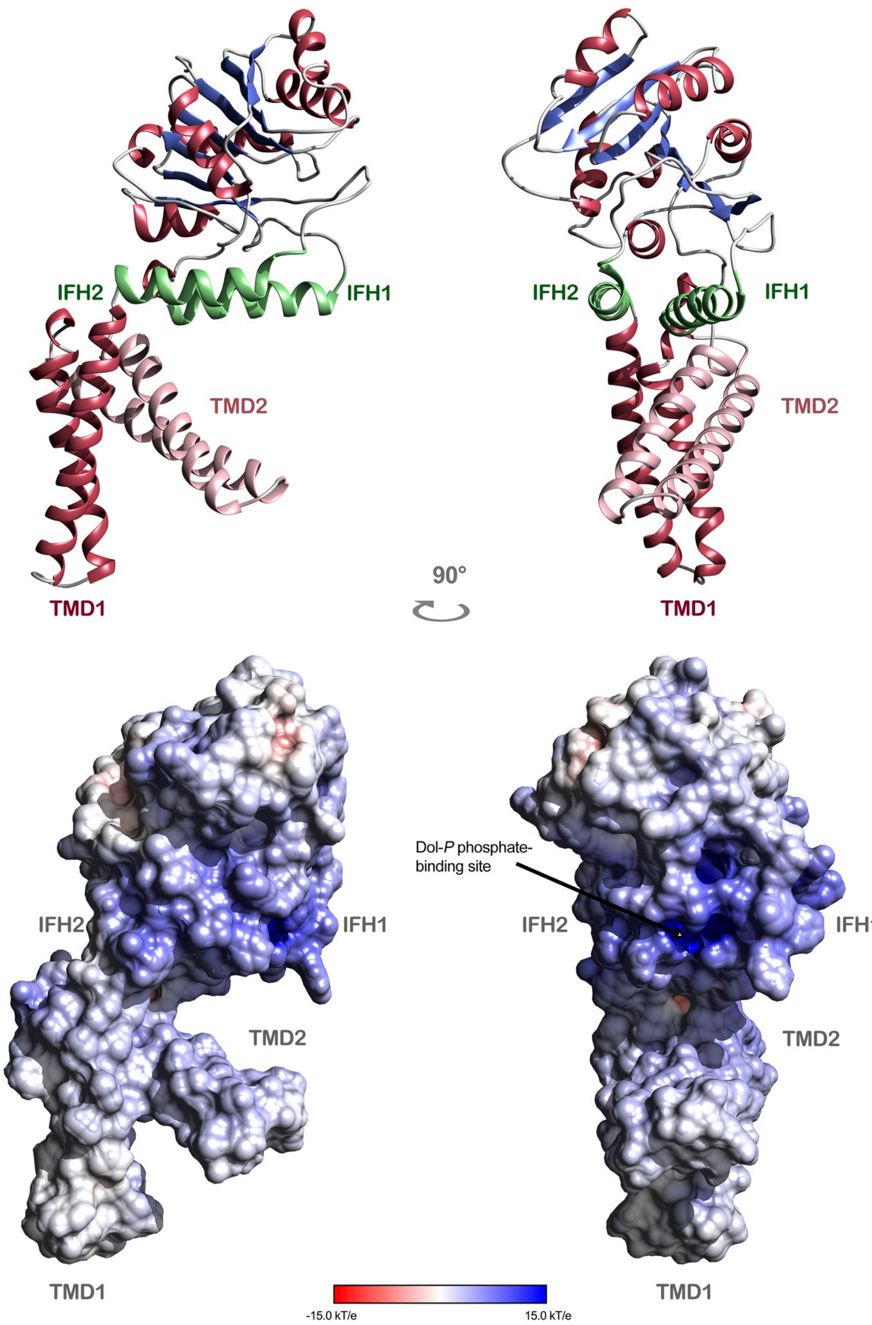

Supplementary Figure 9 | Electrostatic potential surface. Side and front view ribbon representations of *Pf*DPMS (top) and electrostatic potential surfaces (bottom). The electrostatic potential calculated for *Pf*DPMS•GDP-Man•Mn^{2+} complex (omitting ligands) using the PDB2PQR and ABPS functions in UCSF Chimera v. 1.11.2[5], and mapped to a smoothed molecular surface generated with a solvent probe radius of 1.4 Å. PROPKA was used to predict protonation states at pH 7.0. The linearized Poisson-Boltzmann equation was applied, and the system temperature was set to 298.15 K.

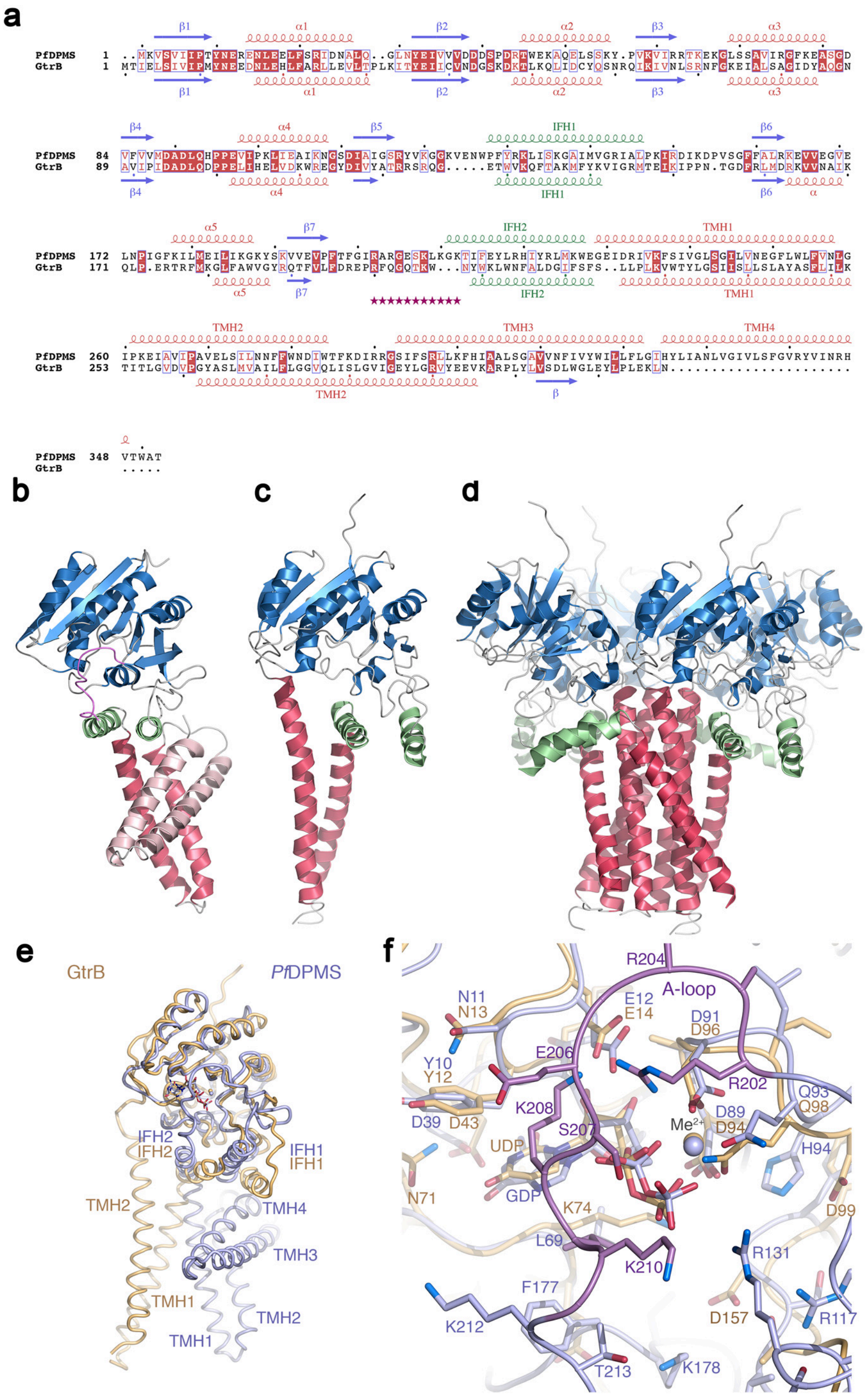

Supplementary Figure 10 | Structural comparison of *Pf*DPMS and GtrB. (**a**) Structure-based primary-structure alignment of *Pf*DPMS and GtrB. The A-loop is highlighted by purple asterisks. (**b**) Overall structure of *Pf*DPMS (GDP•Mg^{2+} complex), (**c**) subunit structure of GtrB, and (**d**) structure of the GtrB homotetramer assembly (PDB code 5EKP[6]). The coloring scheme is the same as used in Figure 1, and the orientation is based on superimposition of *Pf*DPMS and GtrB. (**e**) Superimposition of *Pf*DPMS (blue) and GtrB (yellow) highlighting the different positions and orientations of the IF and TM helices. (**f**) Details of the superimposed active sites in *Pf*DPMS and GtrB. The A-loop (purple in *Pf*DPMS) is disordered and was not modeled in GtrB.

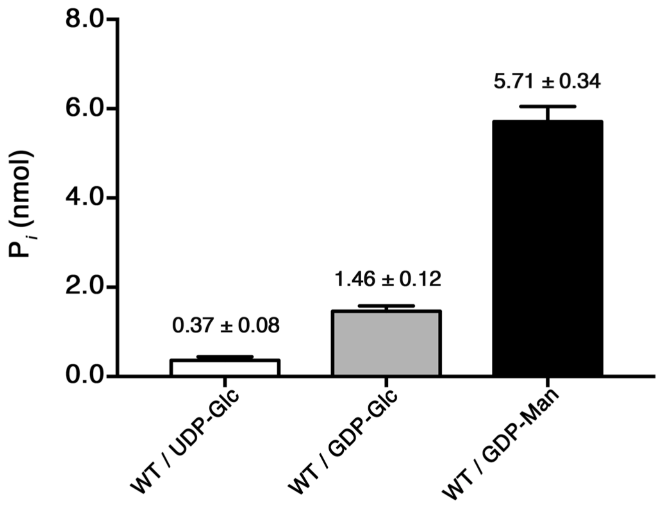

Supplementary Figure 11 | Activity of wild-type *Pf*DPMS using different donor substrates. Amount of product formed measured as nanomoles of released free phosphate using UDP-Glc, GDP-Glc or GDP-Man as donor substrate, and Dol55-*P* as acceptor substrate. Errors are given as mean values ± SEM (*N*=3).

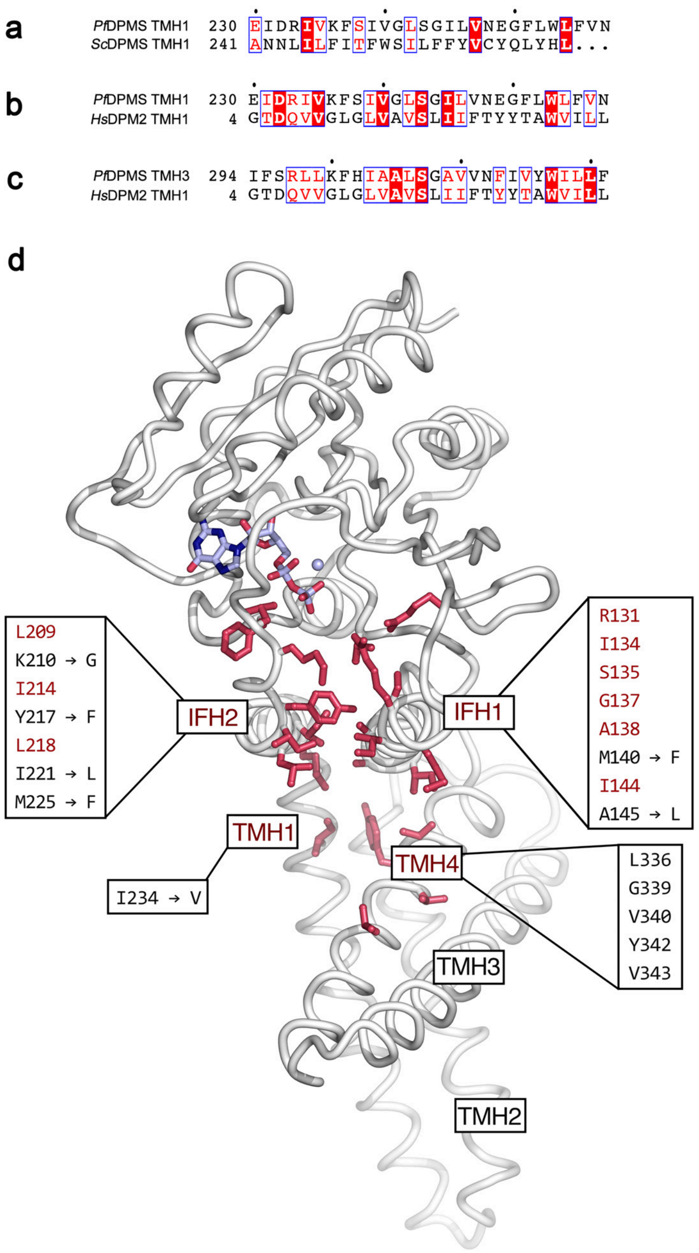

Supplementary Figure 12 | Dol-*P* interactions. Sequence alignment of (**a**) TMH1 in *Pf*DPMS and *Sc*DPMS. The PIRS in *Sc*DPMS corresponds to amino acids 246–258; (**b**) TMH1 in *Pf*DPMS and TMH1 in human DPM2 (UniProt accession O94777); (**c**) TMH3 in *Pf*DPMS and TMH1 in human DPM2. (**d**) Amino-acid side chains that make contact with the dolichyl chain and the phosphate group in Dol-*P* are shown as red sticks (the glycolipid is not shown). The residues were mapped onto the structure of the *Pf*DPMS•GDP•Mg^{2+} complex since the *Pf*DPMS model has a fully built acceptor loop. Within the boxes, red residues are those conserved in *Pf*DPMS and human DPM1. For non-conserved positions, the corresponding replacement is indicated. Alignment of the TMHs in *Pf*DPMS with those in human DPM2 and DPM3 is too uncertain to allow evaluation of possible similarities with respect to the dolichyl-chain interactions observed in *Pf*DPMS.

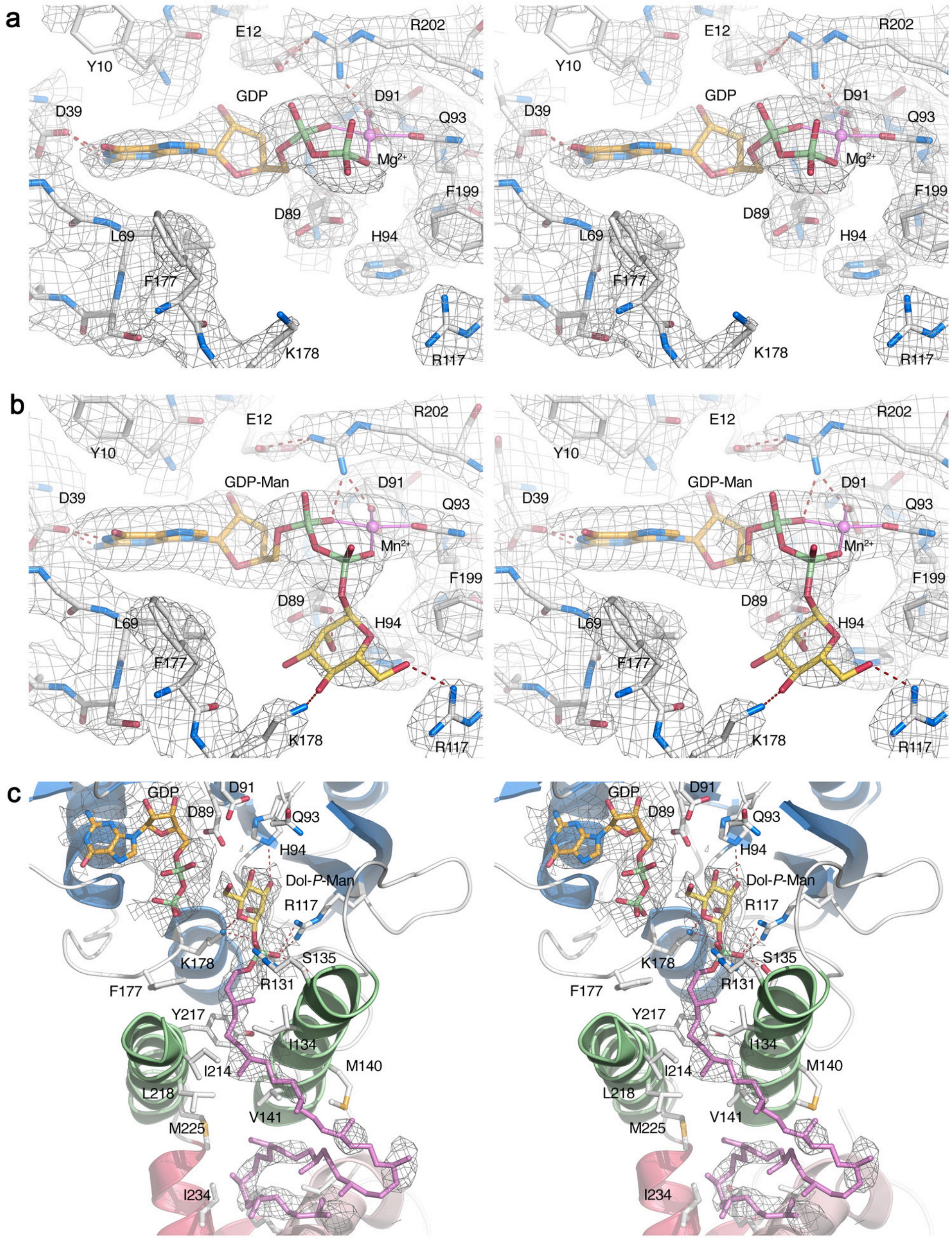

Supplementary Figure 13 | Unbiased electron density for *Pf*DPMS complexes. Stereo views of unbiased electron density covering (**a**) the active site in the GDP•Mg^{2+} complex (5MLZ) with overlaid $2F_o$-F_c map at 0.9σ level, (**b**) active site in the GDP-Man•Mn^{2+} complex (5MM0) solvent-flattened Mn-SAD map at 0.5σ level, and (**c**) the GDP and Dol-*P*-Man products in the complex obtained for the 60°C reaction (5MM1) with GDP-Man and Dol55-*P* ($2F_o$-F_c map at 0.7σ level). Phases used to generate the σ_A-weighted $2F_o$-F_c maps were from models prior to addition of ligands.

Supplementary Table 1 | Data collection, phasing and refinement statistics

	5MLZ (*Pf*DPMS•GDP•Mg^{2+})	5MM0 (*Pf*DPMS•GDP-Man•Mn^{2+})	5MM1 (*Pf*DPMS•GDP•Dol55-*P*-Man)	Derivative 1 GDP•Mg^{2+} / $PbCl_2$	Derivative 2 GDP•Mg^{2+} / K_2PtCl_4	Derivative 3 GDP•Mg^{2+} / merthiolate	Derivative 4 GDP•Mg^{2+} / $PbCl_2$
**Data collection**[a]							
Beamline, λ (Å)	SOLEIL PROXIMA 1, 0.97857	DIAMOND *I*03, 1.8600	SOLEIL PROXIMA 1, 0.97857	SOLEIL PROXIMA 1, 0.95007	SOLEIL PROXIMA 1, 1.06883	SOLEIL PROXIMA 1, 0.99987	SOLEIL PROXIMA 1, 0.95007
Space group	$C222_1$	$C222_1$	$C222_1$	$C222_1$	$C222_1$	$C222_1$	$C222_1$
Cell dimensions *a*, *b*, *c* (Å)	90.87, 146.21, 95.35	90.67, 146.32, 95.09	90.11, 144.54, 97.76	90.71, 146.42, 95.40	90.51, 146.46, 95.72	90.02, 146.39, 95.87	90.48, 146.93, 95.64
Resolution (Å), nominal	42.95 – 2.00 (2.10 – 2.00)	29.60 – 2.30 (2.40 – 2.30)	48.38 – 2.60 (2.70 – 2.60)	47.7 – 2.50 (2.60 – 2.50)	47.9 – 3.30 (3.40 – 3.30)	45.0 – 2.90 (3.00 – 2.90)	43.1 – 3.00 (3.10 – 3.00)
R_{sym}	0.043 (1.210)	0.065 (1.939)	0.175 (3.535)	0.040 (0.367)	0.267 (0.880)	0.031 (0.112)	0.057 (0.304)
I / s*I*	29.2 (2.2)	20.1 (1.6)	14.0 (1.1)	26.8 (4.9)	8.2 (3.8)	41.2 (17.8)	30.9 (6.3)
Completeness (%)	99.8 (99.5)	98.7 (97.2)	99.6 (99.4)	99.8 (99.6)	99.3 (98.3)	99.5 (96.6)	99.8 (99.8)
Redundancy	13.3 (13.3)	12.7 (12.5)	12.8 (13.2)	6.9 (6.9)	6.5 (6.6)	6.8 (6.7)	6.8 (6.2)
CC(1/2)[b]	1.000 (0.876)	1.000 (0.669)	0.999 (0.541)	1.000 (0.970)	0.993 (0.894)	0.999 (0.996)	1.000 (0.963)
Anisotropy ($Å^2$)[c]; Resol. a*, b*, c* (Å)	27.07; 2.0, 2.0, 2.1	34.03; 2.3, 2.3, 2.6	46.56; 2.8, 2.6, 3.4	45.89; 2.5, 2.5, 2.5	103.38; 3.3, 3.3, 4.3	42.39; 2.9, 2.9, 2.9	39.76; 3.0, 3.0, 3.0
Wilson B factor ($Å^2$)	46.5	65.7	77.2	54.7	80.5	58.2	63.4
**Phasing**							
Resolution (Å)				47.70 – 2.50	47.86 – 3.30	45.01 – 2.90	43.07 – 3.00
Overall anomalous Correlation (%)[d]				12	54	18	58
Number of sites used in phasing				1	1	4	3
PP_{iso} (acen/cen)				0.613 / 0.604	0.007 / 0.005	0.228 / 0.199	0.351 / 0.288
PP_{ano} (acen)				0.215	0.003	0.474	0.098
$Rcullis_{iso}$ (acen/cen)				0.569 / 0.584	0.940 / 1.210	0.861 / 0.868	0.743 / 0.961
$Rcullis_{ano}$ (acen)				0.867	0.641	0.804	0.997
FOM (acen/cen)				0.067 / 0.111			

**Refinement**			
Resolution (Å)	42.95 - 2.00 (2.07 - 2.00)	29.6 – 2.3 (2.38 – 2.30)	48.38 – 2.60 (2.69 – 2.60)
No. reflections	43104	28128	19726
R_{work} / R_{free}	0.235 / 0.251	0.234 / 0.263	0.238 / 0.303
No. atoms			
Protein	2872	2873	2799
Ligand/ion	142	121	99
Water	57	14	0
B-factors			
Protein	61.6	84.7	88.8
Ligand/ion	77.9	96.4	92.7
Water	55.9	76.8	
R.m.s deviations			
Bond lengths (Å)	0.006	0.007	0.008
Bond angles (°)	0.99	1.04	1.21
Ramachandran[e]: allowed, favored, Outliers (%)	99.7, 95.8, 0.28	99.7, 96.0, 0.28	100, 92.0, 0

[a] The outer shell statistics of the reflections are given in parentheses. Shells were selected as defined in *XDS*[7] by the user. Only one crystal was used for each data set.

[b] CC(1/2) = Percentage of correlation between intensities from random half-datasets as given by XSCALE. Values given represent correlations significant at the 0.1% level[8].

[c] Values output from the Diffraction Anisotropy Server[9]; https://services.mbi.ucla.edu/anisoscale/

[d] Percentage of correlation between random half-sets of anomalous intensity differences as given by XSCALE. Values given represent correlations significant at the 0.1% level

[e] As determined by *MolProbity*[10].

Supplementary Table 2 | Examples of *dpm1* mutations causing CDG-Ie

**OMIM entry**	**Var.**	**Mutation in human *dpm1***	**Manifestation at the protein level**	**Corresponding position in *Pf*DPMS**	**Reference**
603503	0001	274C>G transversion	Single replacement mutation R92G	Arg63	Kim *et al*. 2000[11]; Imbach *et al*. 2000[12]
603503	0002	Deletion of base pairs 331-343	Premature termination at Thr110, followed by 44 random residues	Ser81	Kim *et al*. 2000[11]
603503	0003	Deletion of base pair 628C	Premature termination at Met213	Ile183	Imbach *et al*. 2000[12]
603503	0004	Missense mutation, 742T>C transition in exon 9	Single replacement mutation S248P	Glu216	Garcia-Silva *et al*. 2004[13]
603503	0005	Splice site mutation, T>A transversion in intron 4, loss of exon 5	Premature termination His124	His94	Dancourt *et al*. 2006[14]
603503	0006	455G>T transversion	Single replacement mutation G152V	Gly122	Yang *et al*. 2013[15]
603503	0007	100-kb intragenic deletion	302 bp deletion in the transcript		Yang *et al*. 2013[15]

OMIM, Online Medelian Inheritance in Man

References

1. Maeda, Y. & Kinoshita, T. Dolichol-phosphate mannose synthase: Structure, function and regulation. *Biochim. Biophys. Acta* **1780**, 861–868 (2008).
2. Abu-Qarn, M. *et al*. *Haloferax volcanii* AglB and AglD are involved in N-glycosylation of the S-layer glycoprotein and proper assembly of the surface layer. *J. Mol. Biol*. **374**, 1224–1236 (2007).
3. Guan, Z., Naparstek, S., Kaminski, L., Konrad, Z. & Eichler, J. Distinct glycan-charged phosphodolichol carriers are required for the assembly of the pentasaccharide N-linked to the *Haloferax volcanii* S-layer glycoprotein. *Mol. Microbiol*. **78**, 1294–1303 (2010).
4. Gautier, R., Douguet, D., Antonny, B. & Drin, G. HELIQUEST: a web server to screen sequences with specific alpha-helical properties. *Bioinformatics* **24**, 2102–2102 (2008).
5. Pettersen, E. F. *et al*. UCSF Chimera – A visualization system for exploratory research and analysis. *J. Comput. Chem.* **25**, 1605–1612 (2004).
6. Ardiccioni, C. *et al*. Structure of the polyisoprenyl-phosphate glycosyltransferase GtrB and insights into the mechanism of catalysis. *Nat. Commun*. **7**:10175 (2016).

   doi:10.1038/ncomms10175
7. Kabsch, W. XDS. *Acta Crystallogr. D Biol. Crystallogr.* **66**, 125–132 (2010).
8. Karplus, P. A. & Diederichs, K. Linking crystallographic model and data quality. *Science* **336**, 1030–1033 (2012).
9. Strong, M. *et al*. Toward the structural genomics of complexes: Crystal structure of a PE/PPE protein complex from *Mycobacterium tuberculosi. Proc. Natl. Acad. Sci. USA* **103***,* 8060–8065 (2006).
10. Chen, V. B. *et al*. MolProbity: all-atom structure validation for macromolecular crystallography. *Acta Crystallogr. D Biol. Crystallogr.* **66**, 12–21 (2010).
11. Kim, S. *et al*. Dolichol phosphate mannose synthase (DPM1) mutations define congenital disorder of glycosylation Ie (CDG-Ie). *J. Clin. Invest.* **105**, 191–198 (2000).
12. Imbach, T. *et al*. Deficiency of dolichol-phosphate-mannose synthase-1 causes congenital disorder of glycosylation type Ie. *J. Clin. Invest.* **105**, 233–239 (2000).
13. Garcia-Silva, M. T. *et al*. Congenital disorder of glycosylation (CDG) type Ie. A new patient. *J. Inherit. Metab. Dis.* **27**, 591–600 (2004).
14. Dancourt, J. *et al.* A new intronic mutantion in the DPM1 gene is associated with a milder form of CDG Ie in two french siblings. *Pediatr. Res*. **59**, 835–839 (2006).
15. Yang, A. C. *et al*. Congenital disorder of glycosylation due to DPM1 mutations presenting with dystroglycanopathy-type congenital muscular dystrophy. *Mol. Genet. Metab.* **110**, 345–351 (2013).
